# Fellow-Workers and Their Doings

**Published:** 1914-02-14

**Authors:** 


### ROUND THE WORLD.

### Fellow-Workers and their Doings.

[The Editor Invites Early Information and Contributions Marked "Fellow-Workers."]

Dr. C. H. Foley, of Ardrahan, in County Galway, has lately been turning his attention to the treatment of ringworm, and has devised a ready means of dealing with this troublesome condition. The method has the merit of simplicity, being nothing more than the spraying of the affected area with ethyl chloride, after first painting it with tincture of iodine. In a brief account of his successful use of this plan, communicated to the *Lancet* (January 24), Dr. Foley advises the preliminary removal of grease by washing the skin with sodium bicarbonate solution, followed by spiritus ætheris.

Mr. F. N. Doubleday, M.R.C.S., L.D.S., will take the chair at the annual dinner of Guy's Hospital Dental Society, which will be held at the Trocadero Restaurant on February 14. In addition to being president of this Society, Mr. Doubleday is a demonstrator and dental tutor at "Guy's"; four years ago he was awarded the Dental Travelling Studentship of his hospital.

The *Medical Officer* records the following public health appointments: Dr. W. Dunston to be assistant school medical officer to the East Sussex district; Dr. T. B. Stedman, of Selby, to be medical officer of health for Leighton Buzzard; Dr. D. W. M. Turpin, of Alvaston, to be medical officer of health for that district, where he has for some time been public vaccinator and medical officer to the Union; and Dr. R. S. Pearson, of Leighton Buzzard, to be medical officer of health for the Linsdale Urban District.

Dr. W. Lindsay Locke, M.D. Edin., who has lately succeeded Dr. A. C. Jordan as senior medical radiographer to Guy's Hospital, is experimenting with emulsions of barium sulphate in gastro-intestinal radiography. For this purpose, barium salts are said to have the advantage of being less expensive and less distasteful to patients than bismuth compound, but, on the other hand, are not nearly so opaque.

Mr. J. H. Ryffel, B.Sc., the recently appointed director of the new pharmacological department at Guy's Medical School, is devoting his attention to the action of saline purgatives. To this end he is examining the results of intra-vascular perfusion with different solutions in isolated lengths of gut, the peristalsis then observed being compared with that of the rest of the intestine. Mr. Ryffel is an old Cambridge man, but took his science degree at London University, and is well known in chemical and physiological circles.

The new assistant surgeon to the department for diseases of the throat at King's College Hospital, Mr. C. W. M. Hope, is an old Durham student. In London he joined St. Mary's resident staff as anæsthetist some six or seven years ago, and subsequently worked hard in the throat, nose, and ear department there as well as at "King's." Mr. Hope is an M.D. Durh. and F.R.C.S. Eng., and lately has been assistant surgeon to the Throat Hospital, Golden Square.

Dr. W. Bain, M.D., F.R.C.P., of Harrogate, is working at Charing Cross Hospital on metabolism with special reference to gout. It is understood that the results which he is about to publish will be the basis of an entirely new theory of uric acid excretion. Dr. Bain is the editor of a well-known text-book of medical practice, and has written extensively on problems of diet and excretion.

Mr. Neville Langton, for three years second assistant secretary at the London Hospital, is to be congratulated on his recovery from an illness which resulted in the loss of his appendix. He is a nephew of the present chairman of the Royal Free Hospital, and at the "London" is famous for his powers of courtly persuasion over unruly patients and their friends.

Mr. A. H. Tubby, M.S., F.R.C.S., who has just been favoured with election as a corresponding member of the Société Française de Chirurgie, is the well-known orthopædic surgeon whose work has for many years been associated with Westminster and the Royal National Orthopædic Hospitals.

A special course of post-graduate clinics commenced at the Cancer Hospital last week (February 4) with a lecture-demonstration by Mr. Charles Ryall on "Cancer of the Uterus." These clinics will be held on Wednesdays at 5 p.m., and will deal particularly with diagnosis and treatment. Various members of the staff have arranged to contribute on successive weeks as follows: Mr. W. E. Miles, on "The Surgery of Cancer of the Rectum"; Dr. Horder, on "The Value of Combined Treatments in Cases of Inoperable Cancer"; Mr. R. H. J. Swan, on "Tumours of the Bladder"; Mr. Cunning, on "Tumours of the Thyroid Gland"; Mr. C. Rowntree, on "Cancer of the Breast"; Mr. Wilson, on "New Growths of the Testicle"; Dr. Knox, on "The Röntgen Diagnosis of Internal Growths"; and Mr. Leitch, on "Some Problems of Cancer."

Dr. S. R. Thompson, medical officer to Paris Street Workhouse, Bermondsey, and a district medical officer, has proved another victim to the arduous duties public officials are called upon to perform. He has completed twenty years' Poor-Law service, and six months ago was allowed leave of absence. Although a comparatively young man—he is only fifty-one years of age—the long rest has done him no good, and he has been compelled to resign. The Bermondsey Board of Guardians attribute his breakdown in health "to the trying nature of the duties of his office and the constant calls upon him by day and night." His condition has been induced by anxiety and overwork, and the character and nature of his official duties. The Guardians have decided to add seven years to his service for the purpose of arriving at the amount of his superannuation allowance, which will be £149 10s. per annum.

Dr. J. Ramsay, M.D., a former student of St. Bartholomew's Hospital, has been appointed assistant physician to the Blackburn and East Lancashire Infirmary, an institution which he previously served as junior and senior house surgeon.

